# Botulinum neurotoxin type C protease induces apoptosis in differentiated human neuroblastoma cells

**DOI:** 10.18632/oncotarget.8903

**Published:** 2016-04-21

**Authors:** Aleksander Rust, Charlotte Leese, Thomas Binz, Bazbek Davletov

**Affiliations:** ^1^ University of Sheffield, Firth Court, Sheffield S10 2TN, UK; ^2^ Medizinische Hochschule Hannover, Institut für Physiologische Chemie OE4310, 30625 Hannover, Germany

**Keywords:** neuroblastoma, differentiation, botulinum, toxin, apoptosis

## Abstract

Neuroblastomas constitute a major cause of cancer-related deaths in young children. In recent years, a number of translation-inhibiting enzymes have been evaluated for killing neuroblastoma cells. Here we investigated the potential vulnerability of human neuroblastoma cells to protease activity derived from botulinum neurotoxin type C. We show that following retinoic acid treatment, human neuroblastoma cells, SiMa and SH-SY5Y, acquire a neuronal phenotype evidenced by axonal growth and expression of neuronal markers. Botulinum neurotoxin type C which cleaves neuron-specific SNAP25 and syntaxin1 caused apoptotic death only in differentiated neuroblastoma cells. Direct comparison of translation-inhibiting enzymes and the type C botulinum protease revealed one order higher cytotoxic potency of the latter suggesting a novel neuroblastoma-targeting pathway. Our mechanistic insights revealed that loss of ubiquitous SNAP23 due to differentiation coupled to SNAP25 cleavage due to botulinum activity may underlie the apoptotic death of human neuroblastoma cells.

## INTRODUCTION

Neuroblastomas are embryonal tumours arising from developing cells of the sympathetic nervous system and are the most common form of extra-cranial solid tumour in childhood [1]. Current treatment of high risk neuroblastoma consists of aggressive chemotherapy followed by resection of the primary tumour and focal radiotherapy. Treatment is completed with a course of retinoic acid to clear up residual tumour cells [2]. Despite this intensive, multimodal therapy only around 40 per cent of children achieve a long term cure and neuroblastoma remains the second biggest cause of cancer-related deaths in infants [3]. With the introduction of humanised antibodies, immunological approaches targeting neuroblastoma with high specificity are gaining increasing attention [4]. The advantage of precise targeting of neuroblastoma cells can be utilised further by attaching a cytotoxic cargo to antibodies, for example cytotoxic enzymes [5]. Such therapeutic approach holds promise as it enables high levels of potency not possible with small molecule drugs. The common ribosome-inactivating proteins gelonin, saporin and ricin have been investigated when coupled to antibodies against neuroblastoma-enriched receptors, e.g. gangliosides GD2 and GD3 [6-8]. However, a major drawback to this ribosome-inhibiting approach has been off-target toxicity due to non-specific uptake into healthy cells of cytotoxic enzymes [9]. It would be important to uncover other cellular pathways which can be utilised in the treatment of neuroblastomas. Here we investigated the potential vulnerability of human neuroblastoma cells to protease activity derived from botulinum neurotoxin type C.

Botulinum neurotoxins (BoNTs) are produced by bacteria of the *Clostridium* genus and are the most potent neurotoxins known to man [10]. They consist of an enzyme (light chain) and a receptor-binding unit (heavy chain) connected via a disulphide bond. The heavy chain in addition to the neuronal binding domain contains a translocation domain necessary for delivery of the enzyme into the cytosol [11]. The botulinum enzyme is a protease that cleaves so-called SNARE neuronal proteins with exquisite specificity [12, 13]. SNARE is an acronym of soluble *N*-ethylmaleimide sensitive factor attachment protein receptors. The three SNARE proteins form a strong complex needed for the membrane fusion of vesicles however their cleavage may also lead to neurotoxicity [14, 15]. Specifically, BoNT type C mediates neurotoxicity by cleavage and depletion of two SNARE proteins - SNAP25 and syntaxin – both being vital for cell membrane recycling processes [15, 16]. We have recently shown that co-introduction of BoNT type C and type D proteases into naïve neuroblastoma cells can cause cytotoxicity and induction of apoptosis [17]. However, the need to deliver two enzymes into the same cell is not an ideal strategy especially if it involves linking to a therapeutic antibody. Here we show that botulinum protease type C alone is sufficient to cause apoptotic cell death of human neuroblastoma cells, but only following differentiation. Importantly, direct transduction of translation-inhibiting enzymes and the botulinum C protease into retinoic acid-treated neuroblastoma cells revealed stronger potency of the latter suggesting a novel neuroblastoma-targeting pathway.

## RESULTS

### Undifferentiated neuroblastoma cells are resistant to BoNT/C

**Figure 1:**
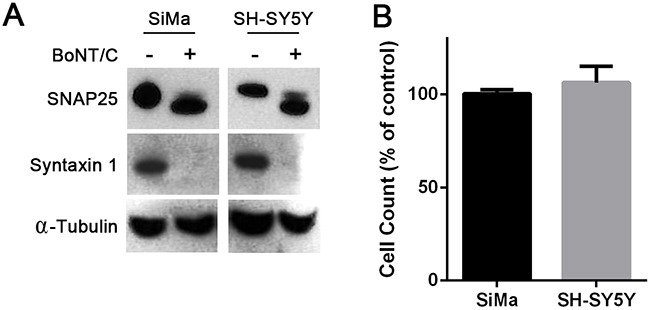
Naive human neuroblastoma cells are resistant to botulinum neurotoxin type C-induced cytotoxicity **A.** Immunoblots showing cleavage of SNAP25 and syntaxin 1 in SiMa and SH-SY5Y neuroblastoma cells following application of BoNT/C (100 nM). BoNT/C hydrolyses 8 and 35 amino acids from the C-termini of SNAP25 and syntaxin1, respectively [16]. Alpha-tubulin was used as a loading control. **B.** Graph showing undifferentiated neuroblastoma cell counts in the presence of 100 nM BoNT/C expressed as a percentage of untreated cells (n=3, mean ± SEM).

The effect of full BoNT/C neurotoxin was first tested on undifferentiated human SH-SY5Y and SiMa neuroblastoma cells [18, 19]. SNARE cleavage in both cell lines was analysed after incubation with BoNT/C at 100 nM for 72 hours. Immunoblotting revealed a massive cleavage of Syntaxin 1 and SNAP25 in neuroblastoma cells (Figure 1A). Despite the observed SNARE cleavage, cells continued to grow normally, as measured by a cell-counting tetrazolium salt-based assay, with no gross cellular abnormalities detected by microscopy (Figure 1B). Thus, when undifferentiated, neuroblastoma cells do not likely require SNAP25 or syntaxin 1 for cell survival and growth.

### Retinoic acid and removal of serum cause robust neuronal differentiation of SiMa cells

Differentiation of SiMa neuroblastoma cells has been shown to greatly enhance their sensitivity to BoNT type A paving the way for a subnanomolar sensitive assay for potency of relevant therapeutic products [18]. We therefore investigated whether differentiation of SiMa cells would also impact on BoNT/C effects. SiMa cells were grown on collagen, laminin or poly-L-lysine for 72 hours either in a serum-supplemented medium or in serum-free medium with B27 supplement. Among the substrates tested, laminin was best to promote a neuronal phenotype and was used for optimisation of differentiation (Supplementary Figure S1A). Addition of retinoic acid, often used to induce neuronal differentiation [20], led to a marked increase in neurite outgrowth and concomitantly reduced cell growth over 10 days confirming terminal cell differentiation (Supplementary Figure S1B, S1C). SiMa cells grown on laminin in serum-free medium in the presence of retinoic acid will henceforth be referred to as differentiated.

**Figure 2:**
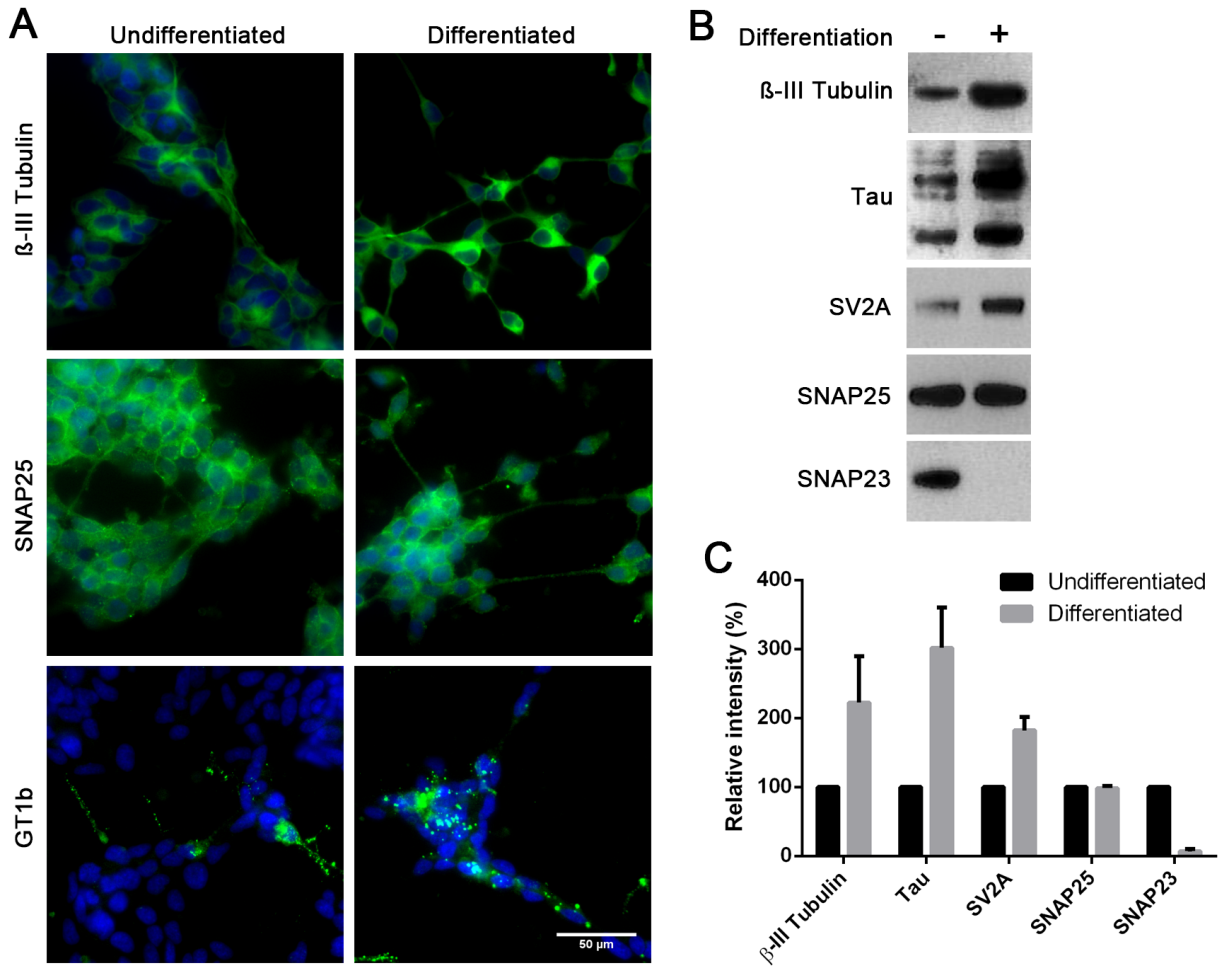
Differentiated SiMa cells exhibit enhanced expression of common neuronal markers and a decrease in SNAP23 content **A.** Immunofluorescent images showing expression of βIII-tubulin, GT1b ganglioside and SNAP25 in SiMa cells before and after differentiation. **B.** Immunoblot showing an increase in expression of neuronal βIII-tubulin, SV2A and tau proteins upon differentiation. SNAP25 content remains stable during differentiation whereas SNAP23 content drops significantly. **C.** Graph showing quantification of immunoblotting signals for indicated proteins (*n*=3, mean± SEM).

To confirm the phenotypic switch of neuroblastoma cells, we analysed the effects of differentiation at a molecular level. Immunocytochemical analysis showed an increase in the BoNT/C binding substrate GT1b following differentiation and the neuronal marker β-III tubulin (Figure 2A). The increase in β-III tubulin levels was quantified by immunoblotting and was shown to be two-fold (Figure 2B, 2C). Immunoblotting also revealed a three-fold increase in the level of the neuron-specific Tau proteins and a two-fold increase in synaptic vesicle glycoprotein 2A (SV2A), a binding target for BoNT type A (Figure 2B, 2C). Taken together, these data confirm strong neuronal differentiation of SiMa cells. No difference in the BoNT type A and C enzymatic substrate - SNAP25 - was observed following differentiation. Crucially, differentiation led to a dramatic decrease in SNAP23, a ubiquitous homologue of SNAP25, to almost undetectable levels (Figure 2B, 2C). SNAP23 is thought to be essential for cell survival [21] and it can substitute SNAP25 in the genetic knockout models thereby preventing neuronal death [15]. This indicates that the depletion of SNAP25 caused by BoNT/C in differentiated neuroblastoma cells may result in cell death.

### Differentiation increases BoNT/C potency and sensitises SiMa cells to BoNT/C-induced cytotoxicity

**Figure 3:**
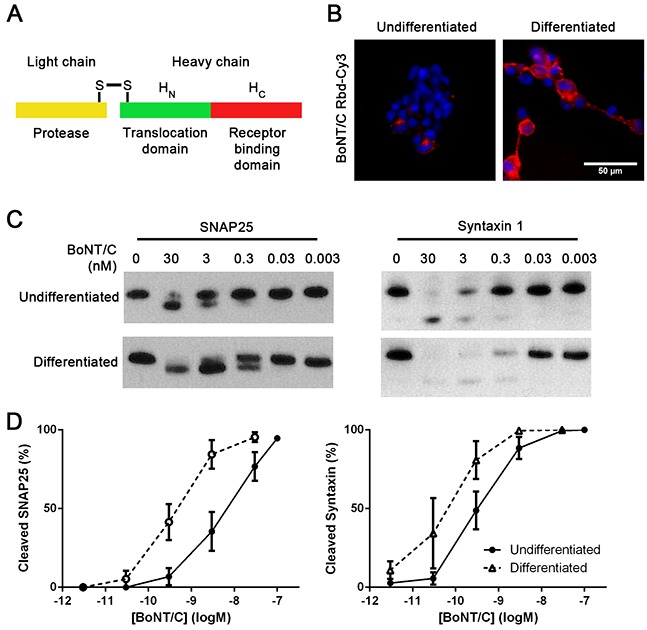
Differentiation of SiMa cells results in enhanced botulinum type C binding and SNARE cleavage **A.** Schematic showing three constituent parts of botulinum neurotoxin. Receptor-binding domain (Rbd, red) can be expressed separately and labelled with a fluorescent dye. **B.** Fluorescent image showing that differentiated SiMa cells exhibit enhanced binding of fluorescent Rbd-Cy3. Immunoblot **C.** and its quantification **D.** demonstrating ten-fold increase in SNAP25 and syntaxin 1 cleavage induced by BoNT/C upon differentiation of SiMa cells (n=3, mean± SEM).

The increased levels of ganglioside Gt1b observed after differentiation of SiMa cells suggested an increase in the level of BoNT/C binding. To confirm this, a BoNT/C Receptor binding domain (Rbd/C, Figure 3A) tagged with Cy3 fluorescent marker was generated by a peptide stapling technique [22]. Live fluorescence imaging showed a marked increase in binding of the Rbd/C-Cy3 following differentiation (Figure 3B) with a ten-fold increase in SNAP25 and syntaxin cleavage by BoNT/C as observed by immunoblotting (Figure 3C, 3D).

**Figure 4:**
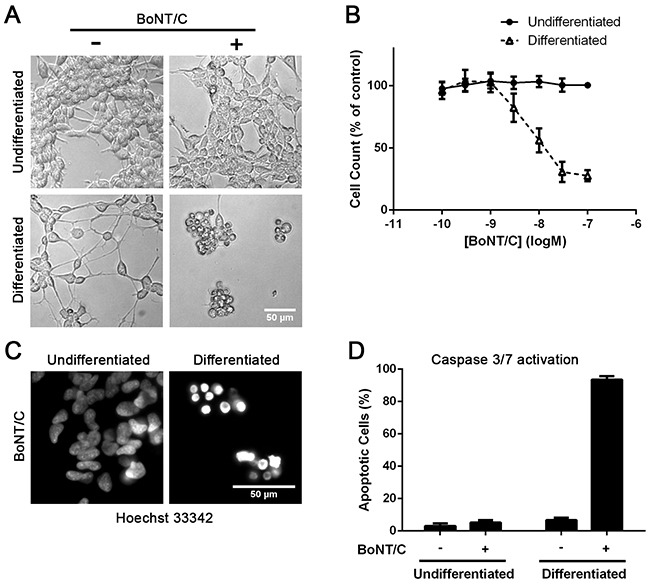
Differentiated SiMa cells undergo BoNT/C-induced apoptotic death **A.** Microscopy images showing dying cells only in the case of differentiated neuroblastoma cells in the presence of BoNT/C (30 nM). **B.** Graph showing dose-dependent cytotoxic effect of BoNT/C on SiMa cells, before and after differentiation, normalised to botulinum-untreated controls (EC50=10 nM, n=3, mean± SEM). **C.** Intensive Hoechst nuclear staining demonstrates apoptosis in the case of BoNT/C-treated neuroblastoma cells as confirmed by a graph showing caspase 3/7 activation (n=3, mean± SEM) **D**.

We next investigated the effect of BoNT/C SNARE cleavage on cell viability. Morphological analysis showed that 30 nM of BoNT/C caused a loss of neurites and cell rounding in the differentiated cells, whereas the undifferentiated cells grew normally (Figure 4A). A large drop in cell number was also observed after differentiation, with an EC_50_ of 10 nM (Figure 4B). Hoechst 33342 nuclear staining revealed that BoNT/C causes nuclear condensation in differentiated cells, indicative of apoptosis (Figure 4C). This was confirmed by a caspase 3/7 activation assay which showed a large level of caspase activation in differentiated cells after BoNT/C treatment (Figure 4D) similar to neuronal apoptosis triggered by BoNT/C [14, 15].

### The BoNT/C protease shows higher cytotoxic potency than translation-inhibiting enzymes

**Figure 5:**
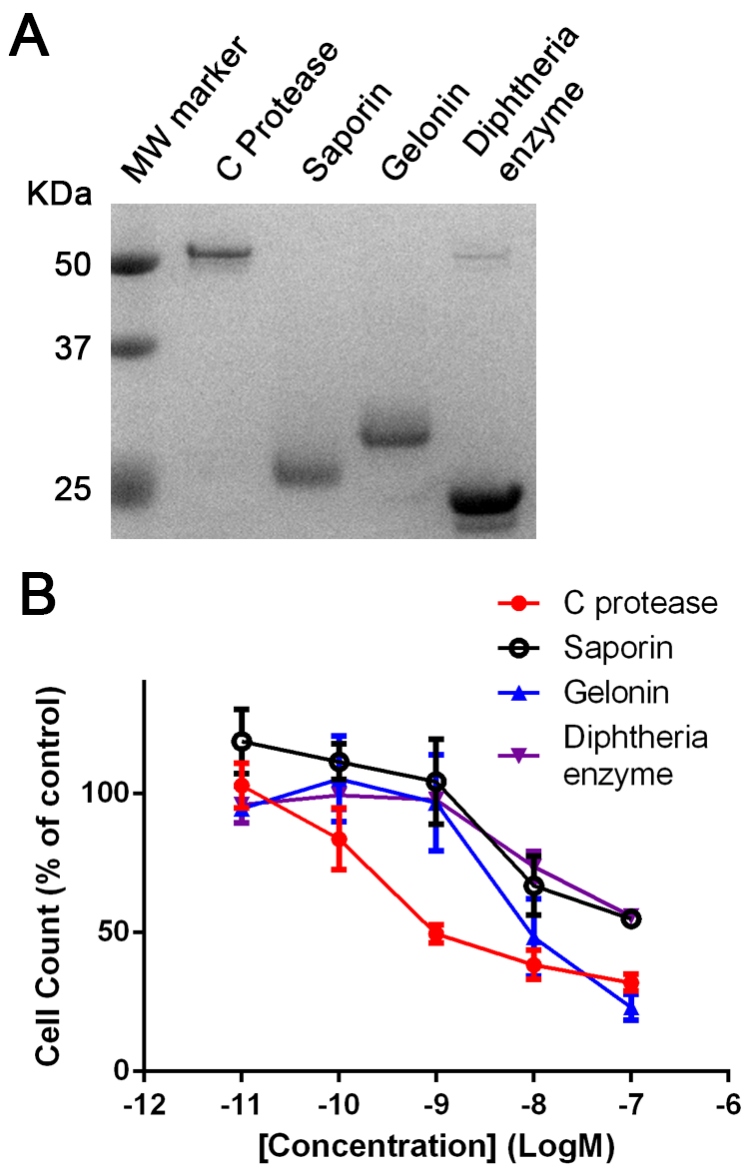
Botulinum type C protease is cytotoxic following intracellular delivery **A.** SDS gel showing botulinum type C protease and the indicated translation-inhibiting enzymes. **B.** Graph showing ten-fold higher vulnerability of differentiated SiMa cells to the botulinum protease (EC50=0.5 nM) in comparison to common translation-inhibiting enzymes. All enzymes were delivered into differentiated SiMa cells using LF3000 lipofection reagent (n=3, mean± SEM). Cell counts were normalised to botulinum-untreated controls (LF3000 alone).

The observed cytotoxic properties of BoNT/C can be utilised in future treatment of neuroblastomas. However, in order to be a viable treatment, BoNT/C would need to show a high level of potency upon entry into cells. A number of translation-inhibiting enzymes including saporin, gelonin and diphtheria toxins exhibit very high potency, meaning that only a small amount of enzyme needs to be introduced into the cell to cause cell death [5]. We have recently shown that enzymes can be efficiently introduced into cells using the DNA transfection reagent lipofectamine 3000 (LF3000) allowing direct comparison of enzymatic cytotoxic potential [23]. We directly compared the efficacy of the BoNT/C protease (i.e. without the Rbd and the translocation domain) against saporin, gelonin and the enzymatic domain of the diphtheria toxin (Figure 5A). Incubation of differentiated SiMa neuroblastoma cells over 72 hours revealed that the BoNT/C protease had at least a 10-fold higher potency than the other enzymes tested (Figure 5B). This suggests that BoNT/C protease is highly toxic upon entry into the cell, making it a promising therapeutic payload against residual neuroblastoma cells.

### Differentiated SH-SY5Y cells also undergo BoNT/C protease-induced cytotoxicity

**Figure 6:**
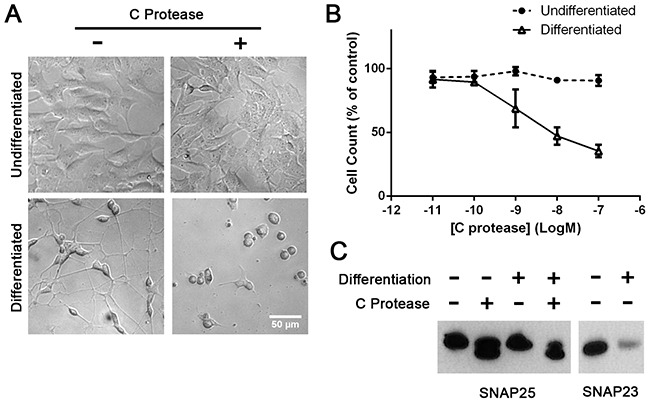
Differentiated SH-SY5Y neuroblastoma cells exhibit sensitivity to botulinum type C protease **A.** The botulinum protease, introduced into cells by lipofection, causes cell death only in retinoic acid-differentiated cells. **B.** Graph showing dose-dependent cytotoxic effect of BoNT/C, before and after differentiation, normalised to botulinum-untreated controls (EC50=1 nM, n=3, mean± SEM). **C.** Immunoblots showing botulinum protease-induced SNARE cleavage and drop in SNAP23 content upon neuronal differentiation of SH-SY5Y cells.

Next we investigated the cytotoxic effects of BoNT/C on widely used human SH-SY5Y neuroblastoma cells. Differentiation of the SH-SY5Y cell line was carried out using the same method as SiMa cells. As with SiMa cells, the BoNT/C protease delivered by lipofection caused a pronounced neurite loss and cell rounding accompanied by a decrease in cell count of differentiated cells (Figure 6A, 6B). Immunoblotting revealed that differentiation of SH-SY5Y cells enhanced SNARE cleavage and led to a significant decrease in SNAP23 content (Figure 6C) confirming that SNARE loss in neuroblastoma cells could be responsible for cell death.

## DISCUSSION

Treatment of neuroblastomas will benefit from introduction of novel agents which can kill required cells with highest possible specificity. Here we demonstrated that differentiation of neuroblastoma cells sensitises them to BoNT/C protease-induced cytotoxicity. Enzymes are currently being investigated for the targeted treatment of cancer owing to their catalytic properties. Conventional enzymes, such as gelonin, saporin and diphtheria toxin block protein translation by inactivating either ribosomes or elongation factors [24]. However, due to the importance of protein synthesis targeted by these enzymes, all cells, both healthy and malignant, are potentially vulnerable with hepatotoxicity and capillary leak syndrome being common [9]. We have shown that the BoNT/C enzyme is significantly more potent than saporin and diphtheria when delivered into differentiated SiMa cells. Such potency makes the BoNT/C protease a possible candidate for targeted therapeutic applications especially because it only cleaves neuron-specific SNAREs. This makes botulinum C protease considerably more specific than conventional translation-inhibiting enzymes. Treatment of neuroblastomas involves the use of retinoic acid after chemotherapy to induce differentiation and stop growth of residual cells [25]. Our results suggest that BoNT/C protease coupled to neuroblastoma-specific antibodies could help to destroy remaining neuroblastoma cells to help prevent relapse. It is worth mentioning that whereas normally the full BoNT/C neurotoxin binds neurons via recognition of complex gangliosides such as GT1b, neuroblastomas express simpler gangliosides GD2 and GD3 which can be efficiently targeted by antibodies [12, 26]. Further work is needed to investigate enzyme coupling and delivery strategies to achieve novel therapeutics for neuroblastomas and this may involve re-engineering of both the C protease and its translocation domain. Recently, an engineered version of botulinum neurotoxin type D was shown to be therapeutically relevant in the treatment of acromegaly opening new avenues for the use of engineered botulinum constructs in medicine [27].

The mechanism by which differentiation sensitises neuroblastomas to BoNT/C protease most likely involves the double loss of ubiquitous SNAP23 and neuron-specific SNAP25 activity. These two SNAREs are known to be necessary for important plasma membrane functions such as exocytosis and membrane recycling [28]. A recent study has shown that knockdown of ubiquitous SNAP23 leads to cell death in a number of cell models, suggesting a vital role for this SNARE in maintaining cell viability [21]. Crucially, exogenous expression of SNAP23 can rescue hippocampal neurons from BoNT/C-induced cytotoxicity [15] demonstrating that SNAP25 and SNAP23 are functionally redundant and only in the case of their simultaneous inactivation apoptosis ensues. The observed cleavage and loss of syntaxin1, and related plasma membrane isoforms [16], likely compound deficiencies in membrane trafficking leading to exquisite sensitivity of neuroblastoma cells to BoNT/C action. The extent of SNAP23 downregulation in neuroblasts following retinoic acid treatment *in vivo* is currently unknown and will be an important future consideration for botulinum-based therapies.

In this study we also demonstrated that under defined conditions SiMa cells can express mature neuronal markers such as Tau and β-III tubulin making this neuroblastoma cell line a useful replacement model for neurobiological studies relying on animal-derived primary cultures. The simple BoNT/C cell death assay using standardised cell cultures can be utilised in detection of this dangerous neurotoxin affecting cattle and poultry, thereby eliminating the current requirement for live rodent bioassays. Future enhancement of sensitivity may involve the addition of GT1b ganglioside for assaying the full neurotoxin as was recently shown for therapeutic BoNT/A preparations [18].

In summary, our study highlights the potential utility of botulinum type C protease in the treatment of neuroblastomas and also sheds light on SNARE function in cell survival which can be exploited in future research and drug development.

## MATERIALS AND METHODS

### Toxins and recombinant proteins

The enzymatic domain of the diphtheria toxin, the BoNT/C protease and the full BoNT/C neurotoxin were described previously [17, 23, 29]. Saporin from *Saponaria officinalis* seeds was purchased from Sigma and gelonin isolated from *Gelonium multiform* was purchased from Enzo Life Sciences. Cy3-Rbd/C was formed in a stapling reaction [20] by mixing SNAP25-Cy3, synaptobrevin-Rbd/C and a syntaxin SNARE helix peptide at equimolar ratios in Buffer A (100 mM NaCl, 20 mM HEPES, 0.4% n-octylglucoside, pH 7.4). SNAP25-Cy3 was prepared by conjugating Cy3-NHS ester to the free cysteine of SNAP25 W105A.

### Cell culture

SiMa cells (DSMZ) were grown in RPMI media (Life Technologies) supplemented with 10% Fetal Bovine Serum (FBS) (Life Technologies). SH-SY5Y cells (Sigma) were grown in a 1:1 mix of MEM (Life Technologies) and F12 nutrient mix (Life Technologies) supplemented with 15% FBS and 1% non-essential amino acids (NEAA) (Life Technologies). Cells were maintained at 37°C, 5% CO_2_. Cell lines were frozen after receiving from supplier and were not used for more than 20 passages (10 weeks).

For differentiation, plates were pre-coated with 10 μg/ml laminin (Sigma) and left at 37°C for at least one hour before washing twice with PBS. SiMa cells were seeded at a density of 1 x10^4^ cells per well in 96-well plates or 2 x10^4^ cells per well in 48-well plates and incubated for 72 hours in differentiation medium (RPMI, 1x B27 (Life Technologies), 1 mM HEPES (Fisher) and 1% NEAA) with or without 10 μM AT-RA (Sigma). SH-SY5Y cells were seeded at 5 x10^3^ cells for 96-well plates and 2 x10^4^ cells for 48-well plates. They were differentiated for 144 hours, using the same differentiation media as SiMa cells, before treatment. 96-well plates with a μClear base (Greiner Bio-one) were used for microscopy.

### Protein transduction

Delivery of the C protease, saporin, gelonin and the diphtheria enzyme into cells using the lipofectamine 3000 (LF3000) reagent (Life Technologies) was carried out as described previously [23]. Protein solutions were prepared in Optimem (Life Technologies) at 10x required concentration with LF3000 (1-2 μl in 100 μl) and then incubated at 20°C for 20 minutes before application to cells (10 μl of protein solution to 90 μl of culture medium).

### Cell count and viability assays

Cells were washed with PBS and then trypsinised. After detachment cells were diluted to 100 μl in media before counting using an automated cell counter (Biorad). The effect of BoNT/C and other enzymes on cell numbers was measured using cell-counting kit 8 (CCK8) assay (Sigma) according to manufacturer's instructions. After treatments, the CCK8 reagent was added to cells in a 1:10 dilution and incubated for 90 minutes at 37°C, before absorbance was read on a plate reader (Biorad) at 450nm. Cell numbers are shown as a percentage of the untreated control after subtraction of the background (media plus CCK8 without cells).

### Microscopy assays

For imaging of BoNT/C Rbd binding, cells were seeded in 96-well μClear plates. Cy3-labelled BoNT/C Rbd (60 ng/ml) was incubated with the cells for 45 minutes at 37°C. 1 μg/ml Hoechst 33342 stain (Life Technologies) was added for nuclear staining. Cells were washed 5 times with PBS before imaging using a digital fluorescence microscope (DMIRB, Leica Microsystems) and a 40x objective. Nuclear morphology was assessed by incubating cells with 1 μg/ml Hoechst 33342 at 37°C for 15 minutes before imaging with a 40x objective. Caspase 3/7 activity was measured using the CellEvent Caspase 3/7 Green detection kit (Life Technologies) according to manufacturer's instructions. CellEvent reagent was added to cells at a final concentration of 5 μM and cells were incubated for 15 minutes at 37°C. Then, 1 μg/ml Hoechst 33342 was added and cells were incubated for a further 15 minutes at 37°C before imaging under a fluorescence microscope at 40x objective. Experiments were performed in duplicate, and at least 4 images were taken per well. Fluorescent cells were counted using ImageJ and apoptosis was calculated as a percentage of total cells. Immunocytochemistry was performed on cells grown in 96-well μClear plates. Cells were washed with PBS, fixed in 4% paraformaldehyde and then permeabilised using PBS-0.1% triton. Cells were then saturated in a blocking solution for one hour before incubation with primary antibodies for 2 hours at 20°C. Primary antibodies were used at the following concentrations: β-III tubulin (R&D Systems, 1:2000), SNAP25 (In-house, 1:500 [30]) and GT1b (1:500). Following washing in PBS, cells were incubated with DAPI stain (Sigma) and the Alexa Fluor 488 conjugated goat anti-mouse or anti-rabbit secondary antibodies (Life Technologies) for 45 minutes. Cells were washed 3 times in PBS before imaging with a 40x objective.

### Immunoblotting

Immunoblotting was performed as described previously [17]. Briefly, cells were lysed in sample buffer (56 mM sodium dodecyl sulfate, 62.5 mM Tris-HCl pH 6.8, 1.6 mM EDTA, 6.24% glycerol, trace bromophenol blue, 1 mM MgCl_2_ and 0.1% benzonase). Protein concentration was measured using the DC assay (Biorad) according to manufacturer's instructions. Lysates were run on 12% Bis-Tris sodium dodecyl sulfate-polyacrylamide gel electrophoresis (SDS-PAGE) gels (Invitrogen) and protein was transferred to a polyvinylidene difluoride membrane (Biorad) before probing with antibodies. Primary antibodies used include SNAP25 (In-house, 1:4000), SNAP23 (Synaptic Systems, 1:4000), Syntaxin 1 (In-house, 1:1000 [30]), SV2A (Synaptic Systems, 1:2000), Tau (1:1000), α-tubulin (1:10 000, Sigma) and β-III tubulin (R&D Systems, 1:1000). Following incubation with peroxidase conjugated sheep anti-mouse or donkey anti-rabbit secondary antibodies (1:24 000, GE Healthcare), proteins were visualised using the SuperSignal West Dura ECL reagent (Thermo Scientific) by X-ray film exposure with signals quantified using ImageJ after film scanning.

## SUPPLEMENTARY FIGURE


