# Supplementary material for: Botulinum neurotoxin type C protease induces apoptosis in differentiated human neuroblastoma cells
Source: Oncotarget. 2016 Apr 21;7(22):33220–8. doi: 10.18632/oncotarget.8903 (PMC5078088; doi:10.18632/oncotarget.8903)
Supplement: Supplementary file 1 [file oncotarget-07-33220-s001.pdf]

SUPPLEMENTARY FIGURE

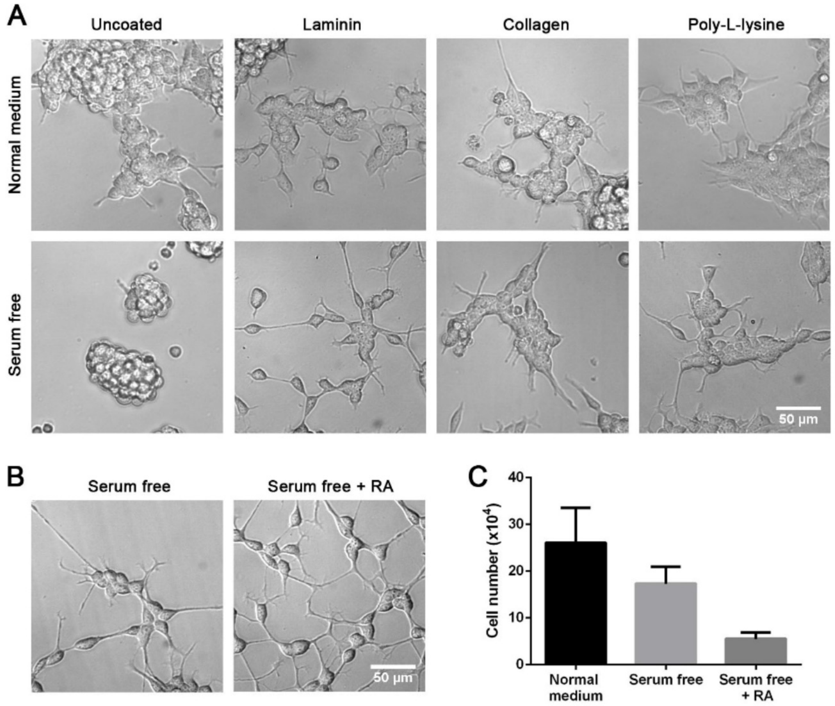

Supplementary Figure S1: Retinoic acid-treated SiMa neuroblastoma cells undergo robust neuronal differentiation. **A.** Laminin provides best support for differentiation of SiMa cells upon serum removal, in comparison to collagen or poly-L-lysine. **B.** Retinoic acid further enhances neuronal differentiation of SiMa cells. **C.** Graph showing decrease in SiMa cell count when cells are grown on laminin and in the presence of retinoic acid, indicating terminal cell differentiation (n=3, mean± SEM).
